# Comparative analysis of postural control and vertical jump performance between three different measurement devices

**DOI:** 10.1371/journal.pone.0222502

**Published:** 2019-09-12

**Authors:** Christopher Blosch, Robin Schäfer, Markus de Marées, Petra Platen

**Affiliations:** Department of Sports Medicine and Sports Nutrition, Ruhr-University Bochum, Bochum, Germany; University of Cassino e Lazio Meridionale, ITALY

## Abstract

**Objectives:**

The aim of this study was to examine the concurrent validity of the HUMAC Balance System (HBS) and Balance Trainer BTG4 (BTG) in comparison to a laboratory-grade force platform (FP) for postural control (PC) and vertical jump performance (VJP) assessment. In addition, reliability of the three devices was measured for PC.

**Methods:**

Overall 22 participants (age = 37.8 ± 13.3 years; gender = 9 male, 13 female; height = 174.1 ± 10.5 cm; body mass = 75.3 ± 17.6 kg) were recruited to participate. Double and single leg standing balance tests with eyes open or closed and counter movement jumps (CMJ) were performed on two separate occasions. Reliability and concurrent validity for COP parameters and VJP were examined using intraclass correlation coefficients (ICC), Bland-Altman plots (BAP), standard error of measurement (SEM) and minimum detectable change (MDC).

**Results:**

COP path length test-retest reliability was predominantly good to excellent for all three devices (ICC = 0.80–0.95). SEM and MDC values were high for all plates (SEM% = 8.0–15.2; MDC% = 22.8–44.5), with the HBS MDC values higher than the KIS and BTG in three of the four trials. ICC scores for concurrent validity were good to excellent for the BTG (ICC = 0.76–0.93) and moderate to good for the HBS (0.49–0.83). Band-Altman plots revealed a systematic bias for the HBS towards higher COP path length values under all conditions and for the BTG in two out of four trials towards lower values. Validity of VJP was excellent for the BTG (ICC = 1.0) and poor for the HBS (0.34), with a systematic bias towards lower values.

**Conclusion:**

The comparative analysis of PC and VJP revealed reliable and valid results for the BTG in comparison to a laboratory-grade force plate. The HBS showed reliable results for PC assessment with restrictions regarding its validity. Results of VJP showed that the HBS revealed deficits in the assessment of activities that require rapid, high force movements such as jumping and running. Due to the variable results of all three devices, it is recommended not to use them interchangeably.

## Introduction

The postural control system guarantees the maintenance of balance and body orientation in the standing position. It is a complex construct that depends on the functions of the nervous, sensory and motor systems [1]. Vertical jump performance (VJP) is used for the assessment of complex motor performance and the lower limb muscle strength/power, especially for the leg extensor chain. Both motor functions are essential for the performance of sports and even activities of daily living. Assessment of postural control (PC) and VJP has been shown to provide valuable information in a variety of situations. Fields of application are e.g. the prediction of falls, quantification of non-specific low back pain, ankle instability and further issues in physical therapy, medicine or engineering [1–3].

But the inherent complexity of the close interaction between sensory perception and motor output makes the comprehensive evaluation of PC very complicated [4]. A large number of different qualitative and quantitative methods for the assessment of PC and VJP have been developed [4]. The qualitative methods use clinical tests, e.g. the Romberg-test or the Berg’s balance test for PC assessment and the jump and reach test for VJP. They show to provide valuable information, but suffer from limitations including subjectivity of scores and the ceiling effect and may thus not differentiate subtle changes [5]. Detailed evaluation of PC or VJP and associated strategies require the use of instrumented tests with several materials to enable kinetic, kinematic, and electrophysiological analysis [6]. Of this quantitative methods force platforms (FP) are the most widely used devices in assessing postural function or VJP [6–8].

FP typically measure ground reaction forces and moments, which are used to calculate force development and position of the center of pressure (COP) [9]. The COP is the origin of all ground reactionary forces in the transverse plane and reflects the neuromuscular response to movements in the center of gravity (i.e. the vertical projection of the center of mass) [4,9]. It is the most frequently used parameter from which various variables can be calculated to assess PC [6]. Most common variables are total COP path length (PL), 95% COP area and COP velocity. PL is defined as the total distance traveled by the COP over the course of the trial duration and is known to be a reliable and valid measure of standing balance [10,11]. 95% COP area is the smallest ellipse containing about 95% of the COP points. COP velocity is the COP excursion divided by the time of the trial and represents the efficiency of the postural control system (the smaller the velocity, the better the PC) [6]. Latter is considered to be the variable with the greatest reliability among trials [1,6].

During vertical jump, the body is pushed upwards against gravity by powerful ground reaction developed as a result of muscular contraction. Jump performance is quantified by the jump height achieved, which can be calculated from the measured ground reaction forces of the FP. Possible formulas are the flight-time-method or the impulse-momentum-method [12]. Most common used jumps are the counter movement jump (CMJ), squat jump or drop jump, which are considered to be reliable and valid in measuring VJP [13].

Based on their objective quantification, force plates are generally considered as the “gold standard” in assessing PC and VJP [6–8]. The HUMAC Balance System (HBS), originally being part of a video game device (Wii Balance Board (WBB)) is increasingly applied as an inexpensive, portable and widespread available force plate in the rehabilitation and assessment of PC and VJP [14–18]. Several studies reported that the WBB is a valid and reliable tool capable of objectively assessing postural stability and VJP [7,19–21]. However, other studies suggest that the WBB may be useful for low-resolution measurements, but should not be considered as a replacement for laboratory-grade force plates [22,23]. To the best of one’s knowledge, there is only one study of Yamamoto *et al*. [20], which investigated the WBB in connection with assessment of jumps. However, they did not measure jump height instead they only measured peak ground reaction force. In addition VJP data of the HBS were partly unsatisfactory.

For further research another measurement device, the 2001 Balance Trainer BTG4 (BTG) was acquired. To date, there are no known evaluation studies for this device.

The purpose of the current study was to explore reliability and concurrent validity of the HBS and BTG compared to an applied valid laboratory-grade force platform (Kistler) to test PC and VJP.

## Materials and methods

### Participants

Twenty-two participants (10 sports science students; 12 adults between the age of 40–60; age = 37.8 ± 13.3 years; gender = 9 male, 13 female; height = 174.1 ± 10.5 cm; body mass = 75.3 ± 17.6 kg) were recruited to participate. In order to exclude any health impairments, all subjects underwent a general anamnesis prior to the start of the study. Potential participants were asked by the investigator regarding current and previous injury history. None of the participants reported any medication intake, current pain, current balance problems, vertigo and orthopedic or neurologic health impairments (e.g. hip or knee endoprothesis, ankle sprain, hearing loss or equilibrium organ dysfunction) that may affect single or double limb standing balance and VJP.

The study was approved by the Ethikkommission (EKS) of the Faculty of Sport Science, Ruhr-University Bochum and was conducted in accordance with the Declaration of Helsinki. All participants were informed verbally and in writing of the procedure and purpose of this study, as well as possible risks such as ankle sprains or falls. Prior to their participations all volunteers provided informed written consent.

### Testing equipment

The HUMAC Balance System (CSMI Solutions, Inc., Stoughton, MA), which has a usable surface of 45 cm x 26.5 cm, is mechanically based on the Wii Balance Board (Nintendo, Kyoto, Japan), but differs regarding its communication interface and a manufacturers corresponding software [4]. While the data exchange of the WBB occurs via Bluetooth, the HBS uses a USB connection, which ensures higher sampling frequency and stable data transfer [4]. The 2001 Balance Trainer BTG4 (HURLABS, Tampere, Finland) is a balance testing and training platform, which measures 96 cm x 68.5 cm in size and has four strain gauge-based load sensors, located in the corners, like the HBS. On the other hand, the 60 cm x 50 cm Kistler (KIS) force platform (Kistler 9260AA6, Kistler Instrumente AG, Winterthur, Switzerland) consists of four piezo-electrical transducers, also placed in the four corners of the device, which are able to measure ground reaction forces in medio-lateral (x), anterior-posterior (y) and vertical (z) planes. In contrast to the KIS, the BTG and HBS merely measure vertical ground reaction forces. However, to compare the data, COP calculations for KIS only took the forces along the z-axis into account.

The Kistler force plate has a sampling rate of 1000 Hz. With 100 Hz, the frequency of the HBS is vastly lower than that of the KIS, but sufficiently accurate in COP measurements, where 40 Hz are the minimum recommendation for recording COP in postural sway [24]. The BTG COP data was acquired at a sampling frequency of 100 Hz as well, but the vertical jump height was sampled at 1200 Hz due to the determination by the manufacturer’s software. Taking into consideration the static noise, the sampling frequency of the Kistler were downsampled at 100 Hz and the raw data for each individual sensor were filtered by a second-order Butterworth low-pass filter and a cutoff frequency of 5 Hz following measurement. The results for HBS were processed by the manufacturer’s software using a single-pole low-pass filter. The BTG contains four sigma-delta AD7730 A/D converters, which include two filters, a low pass (sin/x)^3^ and a 22-tap low pass filter. HBS and BTG data were not processed secondary and were taken directly into analysis.

Prior to the examination, the validity of static COP measurement was verified. For static evaluation, a known load was placed at five different positions (P1-P5) on the surface of each device. Positions were determined according to the reference point P5, which was the center of each respective force plate. P1 to P4 were located at the edges, close to the respective corner points. The COP position coordinates were measured over a time interval of 5 seconds and were repeated three times.

### Procedure

The investigation took part in the Department of Sports Medicine and Sports Nutrition of the Ruhr-University Bochum. Environmental conditions such as lighting and noises were kept stable. The three platforms were set up next to each other on a flat and rigid laboratory floor according to the manufacturer’s installation requirements.

**Fig 1:**
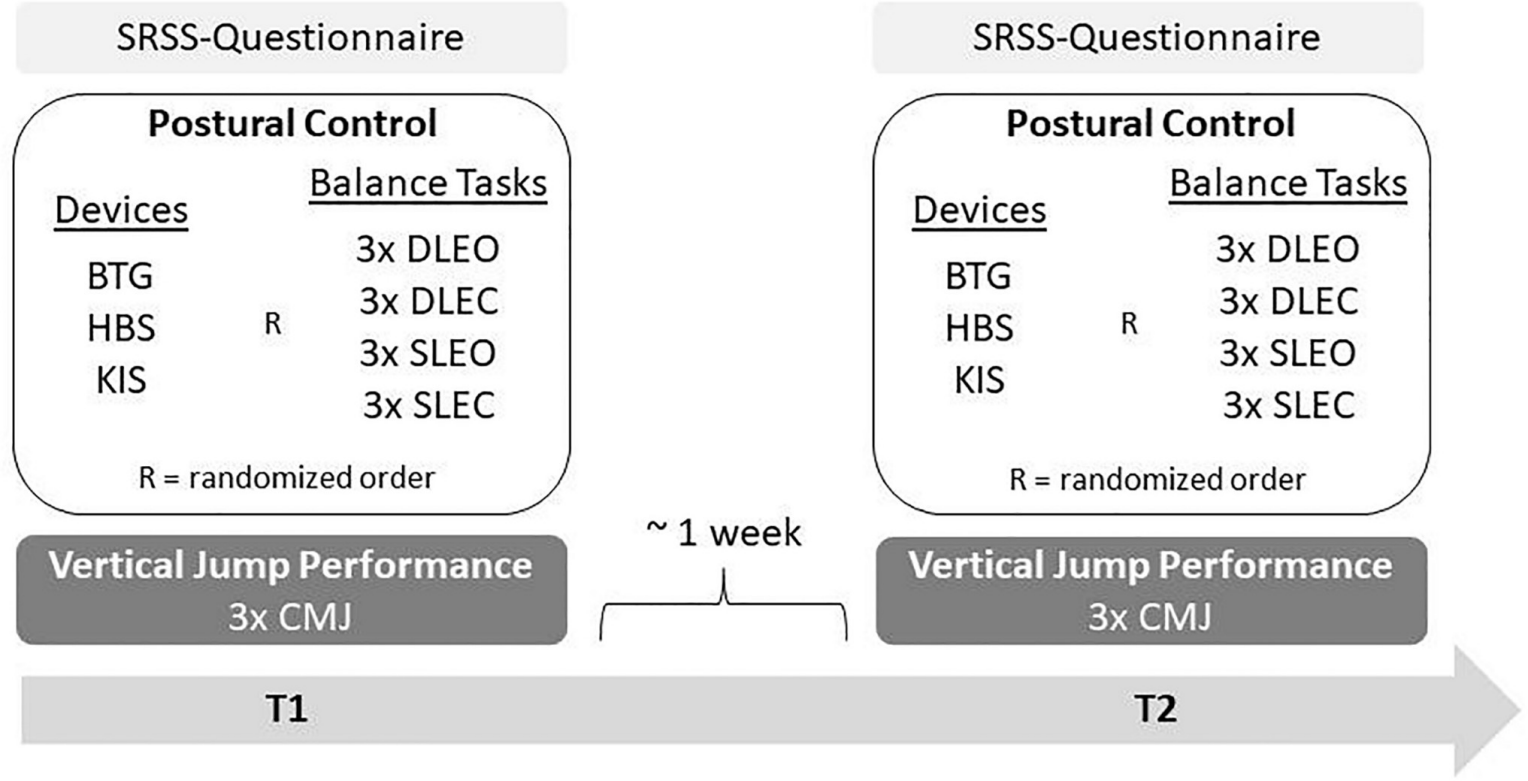
Procedure. BTG = Balance Trainer BTG4; HBS = HUMAC Balance System; KIS = Kistler; DLEO = double limb eyes open; DLEC = double limb eyes closed; SLEO = single limb eyes open; SLEC = single limb eyes closed, CMJ = counter movement jump; T1 = day 1, T2 = day 2.

Participants were tested on two occasions (T1, T2) within 5–8 days (see Fig 1). For postural stability assessment, four standing balance tasks were chosen based on their varying degrees of difficulty and common use in previous literature. These balance tasks were: (1) double limb stance with eyes open (DLEO), (2) double limb stance with eyes closed (DLEC), (3) single limb stance with eyes open (SLEO) and (4) single limb stance with eyes closed (SLEC). Unilateral stance was performed using the preferred leg. The order of (a) standing balance tasks and (b) testing device were randomly assigned for each subject. Participants completed three 30-second trials of each task on each of the three devices, for a total of 36 trials. However, for the elder cohort, duration of the SLEC task was 10 seconds due to the difficulty. All participants were instructed to standard procedure to stand barefoot with their heels and big toes on designated marks, place the hands on the hips, bend the knees slightly and stand as still as possible while focusing a marked point at the nearby wall (distance: 2 m, height: 1.75 m). A trial was considered invalid if participants displaced their standing leg or touched the floor with the contralateral leg during the unilateral stance. Participants received 15 seconds of rest between successive trials within each balance task and 60 seconds of rest when switching between the conditions or devices. Trials were averaged within each condition such that a single value for each task per force plate was obtained. Calculated COP parameters were total COP path length [mm], 95% COP area (95% confidence ellipse) [mm^2^] and COP velocity [mm/s]. Given that the trials were for a fixed time interval, PL and COP velocity are analogous for DLEO, DLEC and SLEO.

VJP of each participant was evaluated by using the counter movement jump (CMJ). To enable simultaneous measurement of data and to eliminate within subject variability, the three devices were placed on top of each other [9,20]. Due to the different sizes, the KIS was placed on top of the BTG and the HBS on the top of the KIS. Whilst each participant performed the standing balance tasks, CMJs are, for security purpose, performed by sport science students only. Vertical jump height was measured for three CMJs per participant and every jump took into account the comparison. The recommended overload capacity was 150 kg for the WBB and 300 kg/sensor for the BTG. Hence, the subjects were instructed to perform submaximal jumps in order to not exceed the loads and occur damage to the measurement devices. Thus, the results of this section are mainly representative for the observed data and comparable to similar flight times/jump heights. Jump height was calculated using the flight-time-method.

Prior to every test day the participants were asked to fill in the SRSS questionnaire (Short Recovery and Stress Scale for Sport) to evaluate the acute recovery and stress state.

### Statistical analysis

A two-way, random-effects, single measure intraclass correlation coefficients (ICC (2,1)) model was used to assess reliability and concurrent validity. Point estimates of the ICCs were interpreted as follows: poor (0–0.39), moderate (0.40–0.74), good (0.75–0.89) and excellent (0.90–1) [5].

Bland-Altman plots (BAP) were used for descriptive evaluation of the concordance of the results from two devices. Specifically, this was performed by plotting the difference for analysed variables between two instruments against the mean results [25]. The BAP shows a possible systematic bias in the differences of the measurements and their mean values. Limits of agreement (LoA) can be calculated by multiplying the standard deviation (SD) of the mean difference of the scores by 1.96 and can predict the difference value of any other measurement by the two devices (with 95% certainty).

To quantitatively describe the within-device test-retest reliability, the standard error of measurement (SEM) and the minimum detectable change (MDC) were calculated. The SEM is the standard deviation of all errors in one measure and represents the absolute reliability. MDC defines the limits within a change in the measurement score that could be attributed to measurement error [26]. SEM was calculated by dividing the standard deviation of the mean differences between the two measurements by the square root of two (SD differences/√2). SEM% and MDC were calculated using following formulas:

$$SEM\%=(SD\times \sqrt{1-\text{ICC}}\times \frac{1}{mean})\times 100$$

$$MDC=1.96\times SEM\times \sqrt{2}$$

In the descriptive approach, the data are expressed as mean value and standard deviation and differences are described as percentage of the deviation. The Kolmogorov-Smirnov test was used to check the normality of the distribution. Correlations between the devices were calculated using Spearman’s rho (rs). Odds ratios are reported with 95% confidence intervals. Statistical significance was defined as a p value ≤0.05.

## Results

### Static measurement

The results of the static COP measurement are presented in Table 1.

**Table 1:** Mean error and SD for the static condition in x- and y-direction.

	Device	P1 [mm]	%	P2 [mm]	%	P3 [mm]	%	P4 [mm]	%	P5 [mm]	%
**X**	BTG	-5.1 ± 0.1	2.0	-5.2 ± 0.8	2.0	3.6 ± 0.3	1.3	-18.4 ± 3.3	7.1	1.3 ± 0.0	1.2
	HBS	-13.0 ± 0.1	6.9	-18.7 ± 1.9	9.9	-10.3 ± 1.2	5.4	-16.5 ± 0.1	8.7	9.3 ± 0.1	9.3
	KIS	-32.2 ± 5.9	12.8	-37.2 ± 9.0	14.9	35.7 ± 3.6	14.3	28.2 ± 6.1	11.3	8.7 ± 2.0	8.7
**Y**	BTG	-17.1 ± 0.1	8.1	21.2 ± 5.4	10.1	-12.1 ± 0.1	5.8	-10.4 ± 2.9	5.0	-9.1 ± 0.1	9.1
	HBS	-10.4 ± 0.2	9.2	-7.5 ± 0.1	6.6	-8.7 ± 0.2	7.7	-7.2 ± 0.1	6.4	-6.9 ± 0.7	6.9
	KIS	-13.4 ± 3.2	6.7	-17.3 ± 1.6	8.7	-15.9 ± 2.6	8.0	-18.4 ± 4.0	9.2	-6.1 ± 1.5	3.1

BTG = Balance Trainer BTG4; HBS = HUMAC Balance System; KIS = Kistler; P = position; X = x-direction; Y = y-direction.

### Postural control

One participant was unable to successfully complete three trials of single limb balance and another one did not turn up at T2. Consequently, test-retest statistical analysis for PC was performed on the data for 21 participants, except for the single limb trials, which included data of 20 participants. VJP data of one participant for T2 was excluded due to a measurement error. Thus, 57 jumps were considered in statistical analysis.

Due to similar results, the focus of the analysis is on the outcomes of the total COP path length. Results of COP area and COP velocity are represented in the appendix (App. A-C).

In general, all three devices showed good to excellent COP path length test-retest reliability (Table 2), except for the HBS under SLEO condition (ICC = 0.72).

**Table 2:** Reliability analysis of total COP path length (mm) measures during each of the four standing balance trials.

	BTG	HBS	KIS
**DLEO**			
**T1 [mm]**	204.1 ± 45.4	293.7 ± 76.8	222.3 ± 54.2
**T2 [mm]**	199.1 ± 45.6	322.8 ± 101.3	229.3 ± 63.1
**Mean Diff [mm]**	-4.8 ± 23.2	25.9 ± 43.2	5.9 ± 32.3
**ICC (95% CI)**	0.87 (0.72, 0.95)	0.85 (0.60, 0.94)	0.85 (0.68, 0.94)
SEM [mm] (%)*	16.4 (8.0)	30.5 (10.1)	22.8 (9.4)
MDC [mm] (%)*	45.5 (22.3)	84.5 (28.8)	63.2 (28.4)
**DLEC**			
**T1 [mm]**	277.2 ± 74.1	445.6 ± 169.4	302.2 ± 91.8
**T2 [mm]**	277.6 ± 75.4	463.4 ± 175.4	318.7 ± 112.0
**Mean Diff [mm]**	1.0 ± 38.9	13.7 ± 101.3	14.6 ± 50.9
**ICC (95% CI)**	0.87 (0.71, 0.95)	0.84 (0.64, 0.93)	0.88 (0.72, 0.95)
SEM [mm] (%)*	27.5 (9.6)	71.6 (15.2)	35.9 (10.5)
MDC [mm] (%)*	76.2 (27.5)	198.5 (44.5)	99.5 (32.9)
**SLEO**			
**T1 [mm]**	902.7 ± 187.4	1216.5 ± 343.1	1030.4 ± 262.7
**T2 [mm]**	857.2 ± 193.5	1101.0 ± 257.5	1026.4 ± 262.6
**Mean Diff [mm]**	-55.0 ± 114.3	-123.5 ± 208.4	-9.8 ± 133.7
**ICC (95% CI)**	0.80 (0.53, 0.92)	0.72 (0.35, 0.88)	0.88 (0.72, 0.95)
SEM [mm] (%)*	80.8 (9.3)	147.4 (14.9)	94.5 (8.8)
MDC [mm] (%)*	224.0 (24.8)	408.6 (33.6)	261.9 (25.4)
**SLEC**			
**T1 [mm]**	1468.5 ± 775.1	1750.3 ± 887.1	1422.1 ± 640.1
**T2 [mm]**	1368.2 ± 766.0	1739.2 ± 875.5	1428.9 ± 676.8
**Mean Diff [mm]**	-136.9 ± 237.3	-54.6 ± 293.9	3.6 ± 251.5
**ICC (95% CI)**	0.94 (0.82, 0.98)	0.95 (0.87, 0.98)	0.93 (0.83, 0.97)
SEM [mm] (%)*	167.8 (12.9)	207.8 (11.3)	177.8 (11.9)
MDC [mm] (%)*	465.1 (31.7)	576.0 (32.9)	492.8 (34.7)

DLEO = double limb eyes open; DLEC = double limb eyes closed; SLEO = single limb eyes open; SLEC = single limb eyes closed; BTG = Balance Trainer BTG4; HBS = HUMAC Balance System; KIS = Kistler; Diff = difference; ICC = intraclass correlation coefficient; CI = confidence interval; SEM = standard error of measurement; MDC = minimum detectable change

*expressed as a percentage of the Day 1 mean value

The SEM (BTG range = 8.0–12.9%, HBS range = 10.1–15.2%, KIS range = 8.8–11.9%) and MDC (BTG range = 22.3–31.7%, HBS range = 28.8–44.5%, KIS range = 25.4–34.7%) values for COP path length were reasonably high for all three devices, with the HBS MDC values higher than the KIS and BTG in three of the four trials.

ICCs for concurrent validity of total COP path length showed good to excellent scores for BTG (0.76–0.93), except for the SLEO condition (0.66). Results of HBS were moderate (ICC = 0.49–0.71) with only good scores for SLEC (0.83) (Table 3).

**Table 3:** Concurrent validity analysis of total COP path length measures during each of the four standing balance trials.

	KIS vs. BTG			KIS vs. HBS		
	Mean Diff [mm]	Mean Diff [%]	ICC (95% CI)	Mean Diff [mm]	Mean Diff [%]	ICC (95% CI)
**DLEO**	-24.1 ± 29.1	10.7	0.76 (0.32, 0.90)	82.2 ± 51.2	26.7	0.49 (-0.10, 0.80)
**DLEC**	-32.9 ± 53.7	10.7	0.77 (0.49, 0.89)	144.0 ± 91.9	31.7	0.52 (-0.10, 0.81)
**SLEO**	-147.4 ± 147.4	14.4	0.66 (0.08, 0.86)	133.0 ± 188.8	11.5	0.71 (0.34, 0.86)
**SLEC**	-3.6 ± 267.4	0.4	0.93 (0.87, 0.96)	324.1 ± 343.6	18.4	0.83 (0.34, 0.94)

DLEO = double limb eyes open; DLEC = double limb eyes closed; SLEO = single limb eyes open; SLEC = single limb eyes closed; BTG = Balance Trainer BTG4; HBS = HUMAC Balance System; KIS = Kistler; Diff = difference; ICC = intraclass correlation coefficient; CI = confidence interval

**Fig 2:**
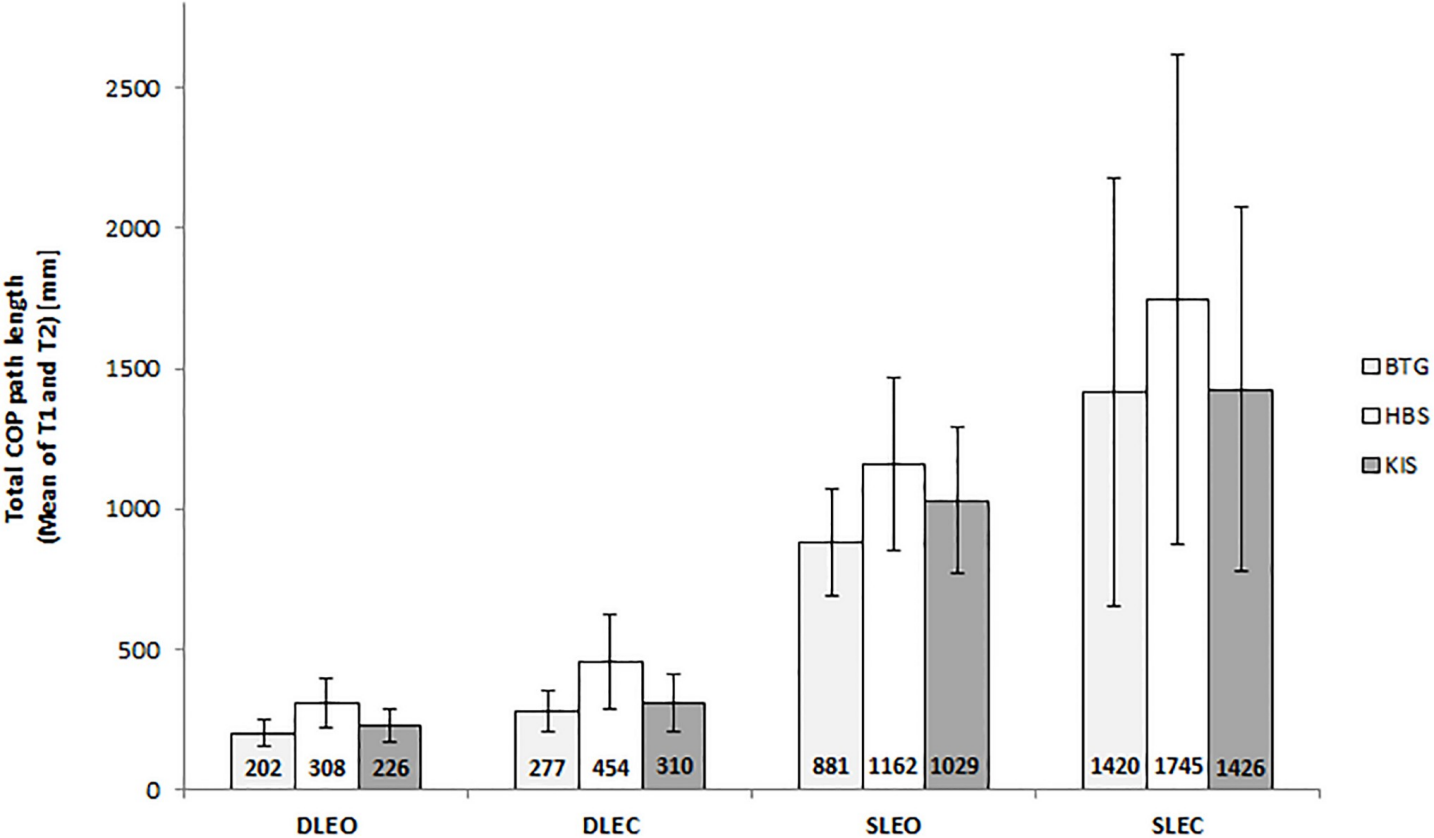
Total COP path lengths (mean of T1 and T2) during each of the four standing balance trials. BTG = Balance Trainer BTG4; HBS = HUMAC Balance System; KIS = Kistler; DLEO = double limb eyes open; DLEC = double limb eyes closed; SLEO = single limb eyes open; SLEC = single limb eyes closed.

**Fig 3:**
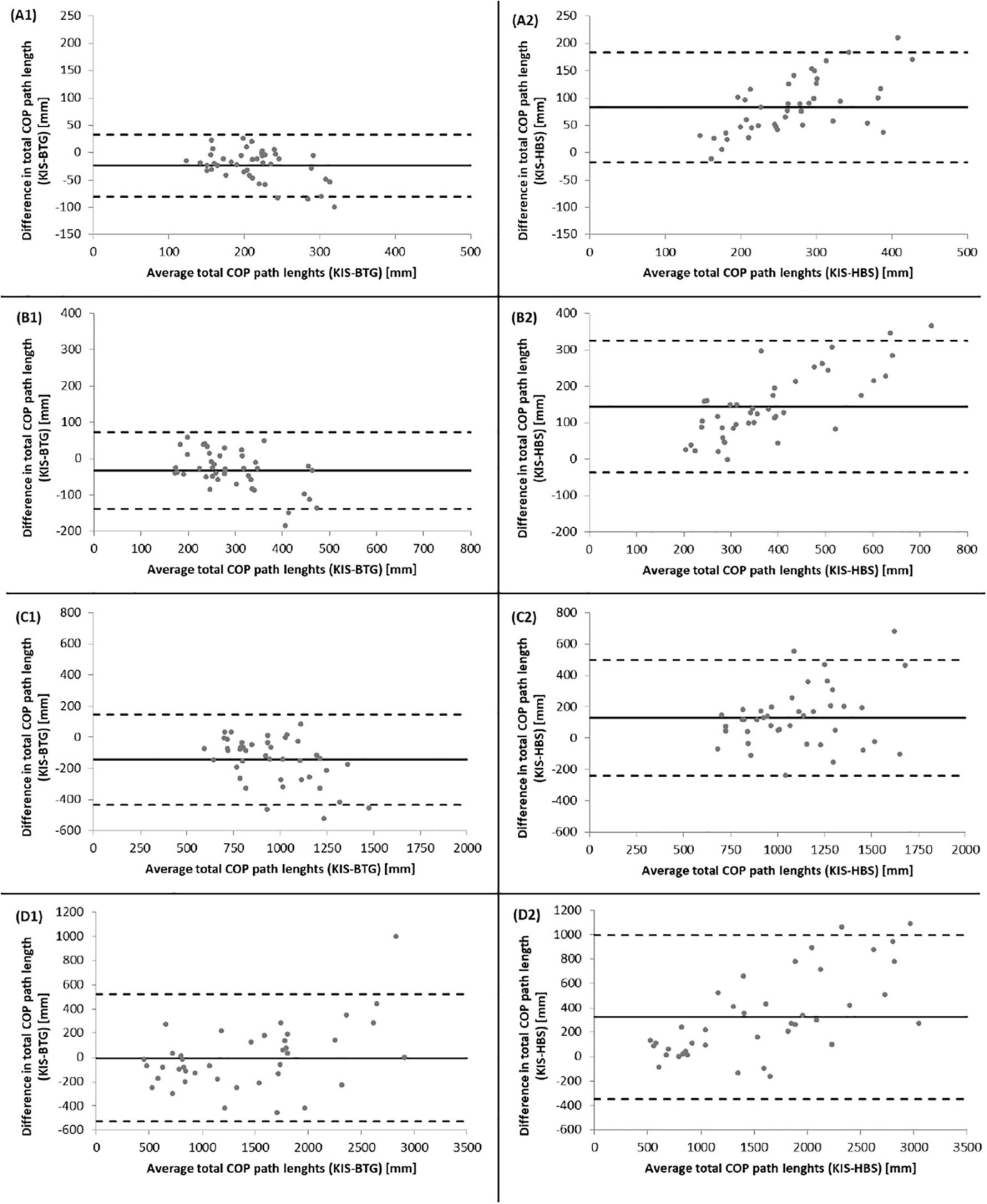
BAPs of total COP path length measures during each of the four standing balance trials. (A = DLEO; B = DLEC; C = SLEO; D = SLEC) between the BTG (1) and HBS (2) to the KIS (^___^ = mean Diff, - - - = LoA). BAP = Bland-Altman plot; BTG = Balance Trainer BTG4; HBS = HUMAC Balance System; KIS = Kistler; DLEO = double limb eyes open; DLEC = double limb eyes closed; SLEO = single limb eyes open; SLEC = single limb eyes closed; LoA = limits of agreement. (*pay attention to the different scaling between standing conditions).

Fig 2 and Bland-Altman plots (Fig 3) demonstrated a systematic bias for the HBS under all conditions towards higher total COP path length values and for the BTG among DLEO and SLEO towards lower values. LOA showed larger variation in the unilateral trials than the bilateral trials.

### Vertical jump performance

**Fig 4:**
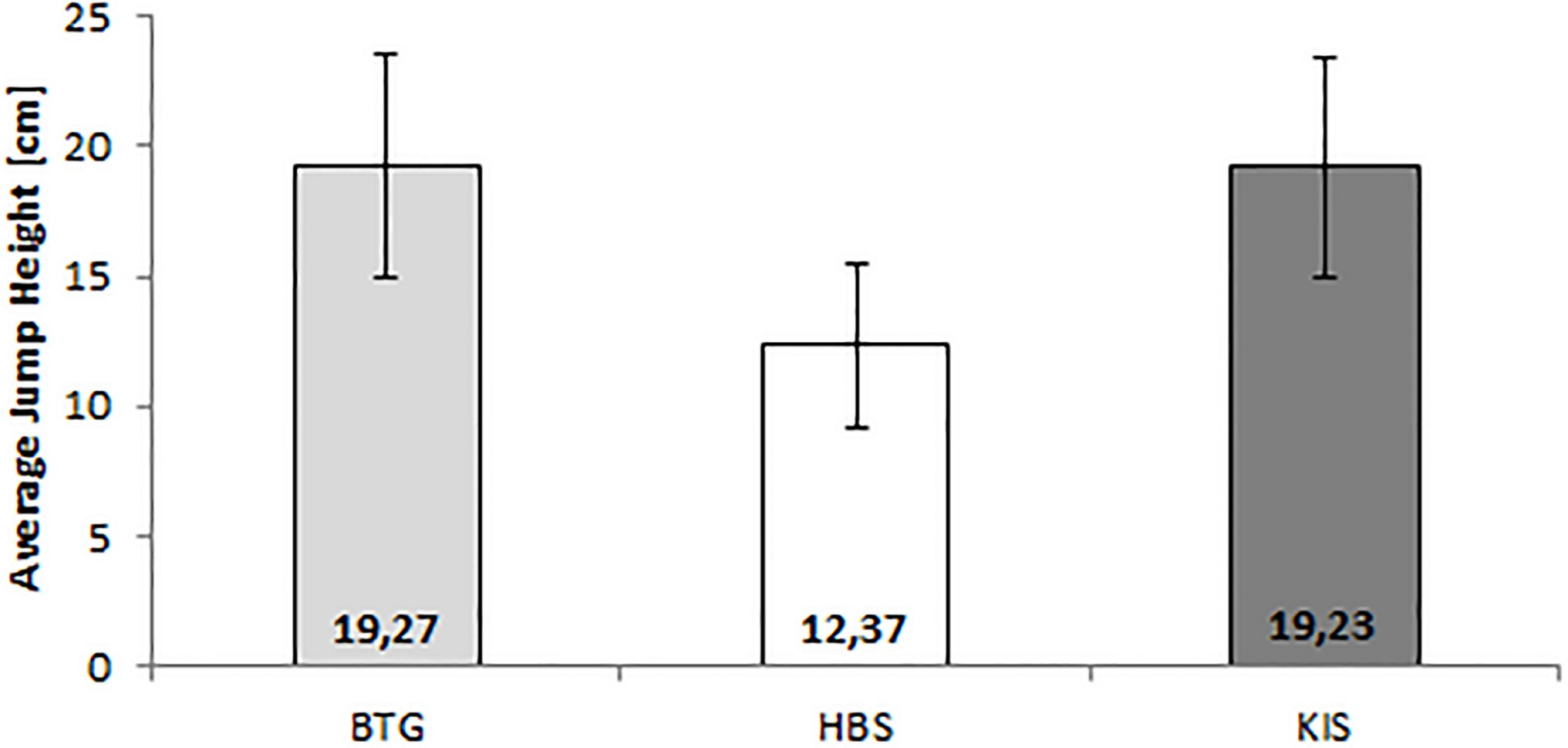
Average jump height between the devices. BTG = Balance Trainer BTG4; HBS = HUMAC Balance System; KIS = Kistler.

**Fig 5:**
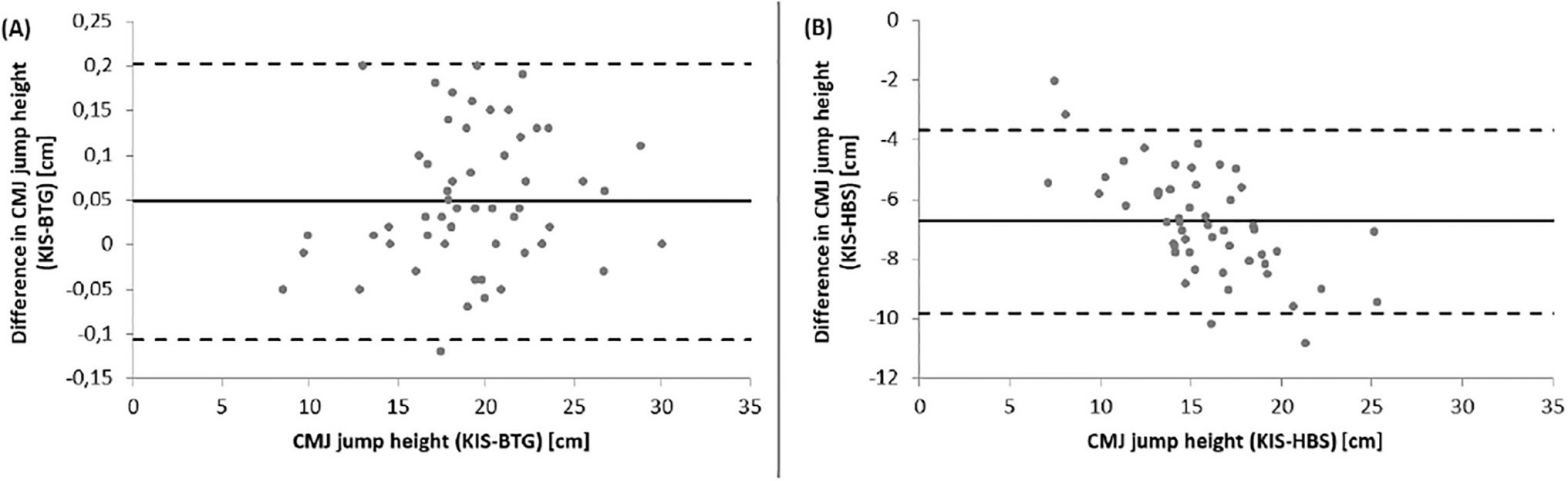
BAPs of VJP between BTG (A) and HBS (B) in comparison to the KIS. BAP = Bland-Altman plot; BTG = Balance Trainer BTG4; HBS = HUMAC Balance System; KIS = Kistler; LoA = limits of agreement. (^**___**^ = mean Diff, - - - = LoA) (*pay attention to the different scaling).

Jump heights of VJP were similar between BTG (19.27 ± 4.27 cm) and KIS (19.23 ± 4.26 cm), but differ a lot to the HBS (12.37 ± 3.18 cm) (see Fig 4). So ICCs for concurrent validity showed an excellent value for the BTG (1.0) and a poor value for the HBS (0.34) (see App. D). BAPs (Fig 5) demonstrated no systematic bias for the BTG and a bias towards lower values for the HBS. The differences increase with jump height for HBS.

**Fig 6:**
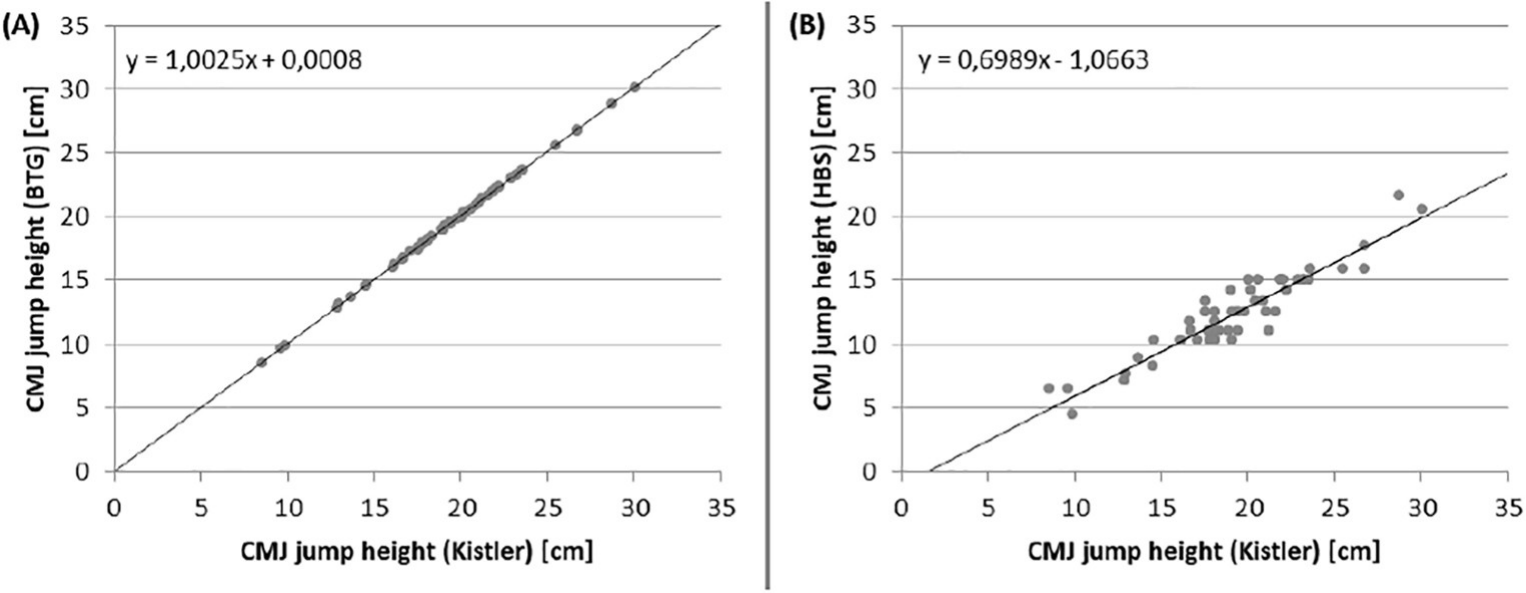
Correlations between jump heights of BTG (1) and HBS (2) to the KIS. BTG = Balance Trainer BTG4; HBS = HUMAC Balance System; KIS = Kistler; CMJ = counter movement jump.

Correlations between both devices to the KIS were significant (p<0.001), with spearman’s rho for the BTG (rs>0.99) higher than for the HBS (rs>0.89). Fig 6 demonstrated a variance for HBS jump heights and the regression line visualise the underestimation of the jumps.

## Discussion

The objectives of the current study were to investigate concurrent validity of the HBS and BTG in comparison to a laboratory-grade force platform for PC and VJP assessment, and for PC to additionally explore reliability of these devices. The ability to objectively assess standing balance and VJP using alternatives to expensive laboratory-grade FPs could provide numerous benefits in sports science in terms of improving athletes or investigating impairments. Our findings suggest that the BTG is a reliable and valid measurement tool capable of assessing PC and VJP. The HBS showed reliable results for PC measurement with deficits regarding its validity but failed to measure VJP.

### Static measurement

Results of the static conditions showed for all devices that the average margin of error were largest near the corner points. Koltermann *et al*. [4] suppose that this phenomenon can be attributed to the positions of the sensors being in each respective corner. The system limits are exceeded by placing the known load directly on the edges of the device. This leads to an increased rate of error at P1 to P4, whereas the error for P5 is lower. The Kistler force plate is based on piezoelectric sensors and is therefore generally considered to manage dynamic conditions well, but shows deficits under static conditions. The devices of the HBS and the BTG are fitted with strain gauges, which register compressing or stretching deformations and, therefore, exceed the performance of the Kistler under purely static conditions [4].

### Postural control

Due to the complexity, balance assessment is not perfectly reliable [2]. But in line with previous studies [2,9,27,28], present ICC results indicated a high degree of agreement between test sessions for all devices (BTG = 0.8–0.94, HBS = 0.72–0.95, KIS = 0.85–0.93). Clark *et al*. [7] compared COP PL of a WBB with a laboratory-grade FP (AMTI Model OR6-5) with the same balance tasks and reported similar reliability (WBB = 0.66–0.91, FP = 0.79–0.94). Clark *et al*. [9] confirmed these results in a review that examined twelve studies regarding reliability of the WBB related to other force plates. The majority of the included studies revealed predominantly moderate to excellent reliability.

The MDC of the BTG and HBS in percentage was relatively high in the current study, but in accordance with previous studies [7,11,26,28,29], and was similar to that of the KIS. These findings demonstrated that reasonably large variations in balance assessment were necessary in test-retest studies to reveal a significant change in performance. Values in excess of 20% indicate that low magnitude changes in PC performance would not be statistically detected by either the KIS, BTG or HBS force plates [7].

In order to exclude the influence of the physical condition on the performance between the measurement dates, the SRSS questionnaire was collected. Results revealed no significant correlation between changes in performance and current recovery-stress state in eleven out of twelve cases (see App. E), so it can be concluded that reliability was not substantial affected.

With regards to concurrent validity, ICC point estimates of the BTG showed good to excellent values except for SLEO. The HBS only received moderate values for DLEO, DLEC, SLEO (0.49–0.71) and a good value for SLEC (0.83). The amount of agreement appears to depend on task complexity. Double limb stance with opened eyes can be regarded as the easiest task and single limb stance with closed eyes (no visual feedback) as the most complex. Thus, lower ICC scores were observed for the easy tasks during bipedal stance and ICCs increased with higher complexity of the task.

Examination of the Bland-Altman plots revealed a systematic bias for the HBS towards higher COP values under all conditions and for the BTG towards lower values in two of the four trials. As discussed in previous studies [7,30,31] it is probable that the different values received between the BTG, HBS and KIS are the result of device-specific factors, such as differences in size, surface texture and hardness or the sensitivity and accuracy of the sensors. Qualitative feedback from participants supports this argument, as several subjects stated that they felt kinesthetically ‘less stable’ on trials performed on the HBS in comparison to those that were performed on the BTG or KIS. This could be related to the fact that the HBS has a much narrower surface than the BTG or KIS (26.5 cm versus 68.5 cm and 50 cm), and its plastic shell being less rigid [30]. The over-estimation of the HBS is consistent with the results of previous studies [5,7,32–35] and appears to be a typical feature of uniaxial force plates [6]. It cannot be safely explained why the BTG underestimates the COP data. It is important to emphasise that we did not analyse the data on the same raw and filtered data level, respectively. Whereas sampling frequencies (100 Hz) were identical between devices after downsampling KIS data, it seems reasonable to assume that HBS and BTG system-immanent raw-data processing may account for the observed COP path length differences.

With regards to the other COP parameter, overall COP area received slightly lower ICC values for reliability and validity, except for the validity of HBS, which increased. Especially reliability analysis of HBS was lower for double limb trials with only poor to moderate ICC values (0.15–0.50). COP velocity showed similar results in comparison to COP PL. Results indicate that COP values do not differ much, but it is important to know which measures are most sensitive to the changes occurring in PC assessment. The parameters used are the most common in the literature and are described as reliable and valid in measuring postural stability.

### Vertical jump performance

**Fig 7:**
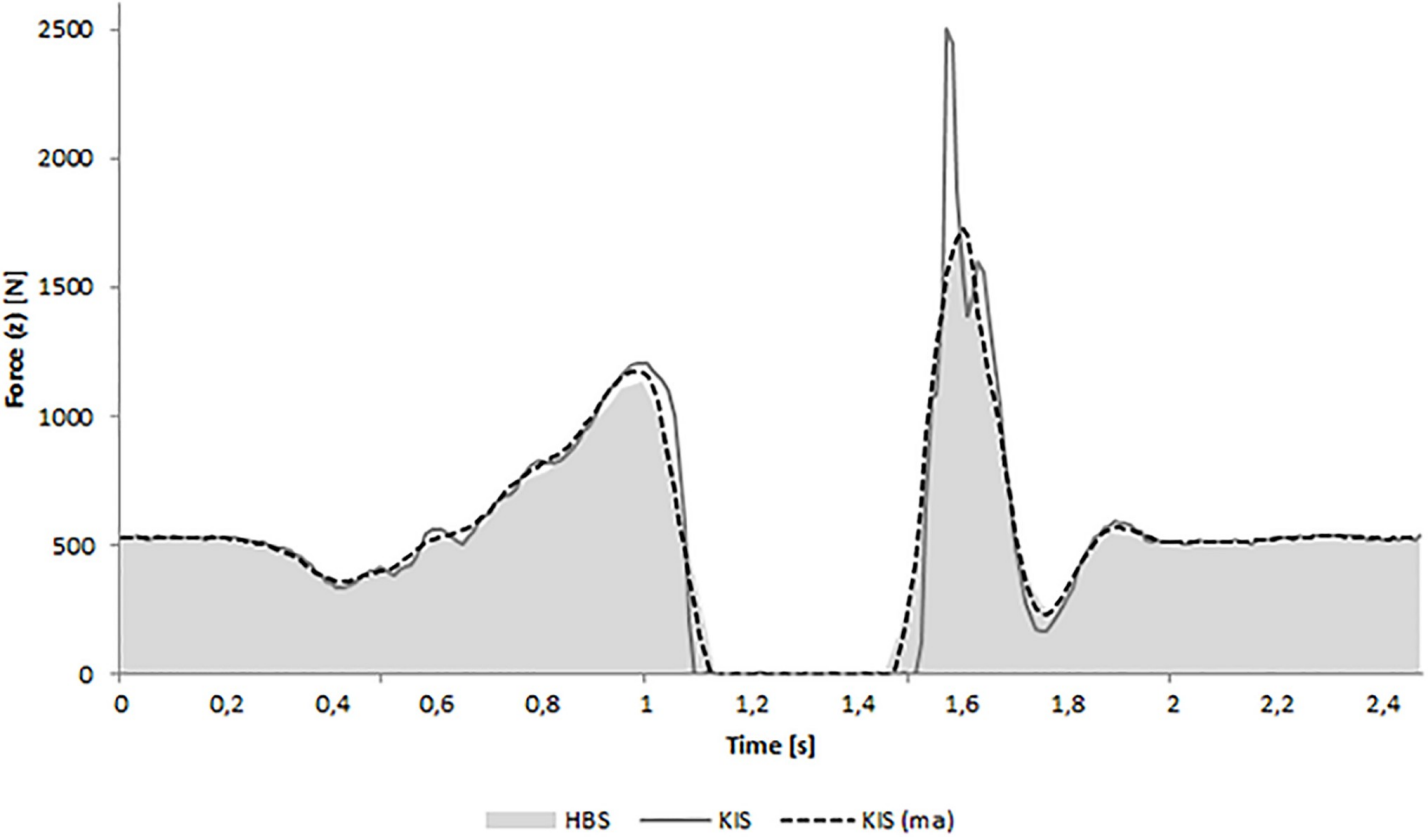
Force-time curves of one identical jump measured from HBS and KIS, and an edited KIS curve with a moving average of 100 data points (KIS (ma)). HBS = HUMAC Balance System; KIS = Kistler.

Results of VJP indicate that the HBS is not capable of measuring the jump height precisely. The mean difference was 35.8% lower compared to the FP and validity was poor (ICC = 0.34). In contrast, the BTG showed very small differences (0.2%) and an excellent validity (1.0). One reason for the failure of the HBS could be the sampling frequency. HBS frequency was much lower (100 Hz) than that of the KIS (1000 Hz) or BTG (1200 Hz) and did not reach to the recommended 1000 Hz for recording CMJ [36]. Beside the low sample rate, the WBB has more limitations compared to a FP like the unavailability of horizontal forces, a larger amount of noise, an inconsistent sampling interval, occasional glitches in the data, and a manufacturer advised maximum load of 1962 N [37,38]. Due to the limitations, Clark *et al*. [7] mentioned that the WBB cannot be a direct replacement of a FP in activities that require rapid, high force movements such as jumping and running. Yamamoto *et al*. [20] investigated the validity of a jump training apparatus using the WBB and reported about nearly comparable data to those of a FP when assessing jumping force. The outcome measure used in his study was the peak vertical ground reaction force during the landing and jumping phase. However, Yamamoto did no further research to determine the jump height. In the current study, jump height was calculated using the flight-time-method. Due to Yamamoto’s results, jump height for KIS and HBS was additionally calculated using the impulse-momentum method. ICC score showed a moderate validity (0.55) and mean difference decreased to 14.9%. These findings indicate that the impulse-momentum method provides better results, but still does not correspond to these of the BTG or KIS. Yamamoto mentioned that the WBB cannot measure peak levels exactly in situations involving strong forces in excess of 1800 N, however usually forces of a CMJ exceed 2500 N during landing [12]. One explanation could be the deformation of this device by the shock of the landing, due to its plastic shell. Another explanation for the differences in the current study compared to Yamamoto could be the data processing of the HBS in contrast to the WBB. Fig 7 shows a comparison of a CMJ between the HBS and KIS. Curves were overlapped and synchronised in relation to the first peak force. Additionally a moving average of 100 data points for KIS is plotted. Curves of moving average and HBS look very similar, so data processing can be a solid explanation.

### Methodical reflection

A limitation of this study is that asynchronous concurrent validity assessment was performed for PC measurement. Clark *et al*. [9] reported that ICC or correlation scores with asynchronous testing were typically lower than those measured with synchronous testing. This is to be expected, as this form of concurrent validation also includes an aspect of within subject variability.

Only validity is assessed for VJP and it would be interesting to see how the reliability of the three different devices looks like. So further research is needed to explore this issue.

A further restriction is that the CMJ was just performed submaximal. According to the findings, differences seem to increase with jump height, so validity would be influenced and decrease further with maximal performance.

As mentioned earlier, it is important to note that it was not possible to get unfiltered data for the BTG and HBS, due to previous system-immanent raw-data processing. Therefore we were not able to do the analysis on the same raw and filtered data level, respectively, so this is the most plausible explanation for the differences between the devices.

## Conclusion

Force plates are useful diagnostic tools for the assessment of the kinetic characteristics of a human’s movement. They provide information about the external forces involved in movement to qualitatively evaluate the human’s execution of a skill or physical development. In this connection, PC and VJP are two areas of application where force plates are generally considered as the “gold standard”. However, they are often expensive, cumbersome to transport and difficult to setup and therefore not feasible in a clinical setting. So alternatives to laboratory-grade force plates are wanted. Results of the present study indicate that the BTG is a reliable and valid measurement tool capable of assessing PC and VJP. The HBS may be useful for measuring vertical ground-reaction forces and COP with limitations on accuracy and precision. Rapid and high force movements as in VJP measurement are not able to be evaluated sufficiently. Therefore HBS should not be considered to be equivalent to laboratory-grade equipment. However, HBS may provide an estimate of force and COP measures that could be useful for situations where lower accuracy and precision is acceptable. In summary, also the data of the BTG vary from data of the KIS, thus measurements of the evaluated devices should not be used interchangeably.

## References

[1] Duarte M, Freitas SM. Revision of posturography based on force plate for balance evaluation. Rev Bras Fisioter. 2010; 14(3): 183–92. 20730361

[2] Ruhe A, Fejer R, Walker B. Center of pressure as a measure of balance performance in patients with non-specific low back pain compared to healthy controls: a systematic review of the literature. Eur Spine J. 2011; 20: 358–68. 10.1007/s00586-010-1543-2 20721676 PMC3048236

[3] Mueller S, Mueller J, Stoll J, Cassel M, Hirschmüller A, Mayer F. Back pain in adolescent athletes: results of a biomechanical screening. Sports Medicine International Open. 2017; 1: E16–E22. 10.1055/s-0042-122713 30539081 PMC6226062

[4] Koltermann JJ, Gerber M, Beck H, Beck M. Validation of the HUMAC balance system in comparison with conventional force plates. Technologies. 2017; 44(5): 1–12.

[5] Zakeri L, Jamebozorgi AA, Kahlaee AH. Correlation between center of pressure measures driven from wii balance board and force platform. Asian J Sports Med. 2017; 8(3): e55436.

[6] Paillard T, Noé F. Techniques and methods for testing the postural function in healthy and pathological subjects. BioMed Research International. 2015; 2015: 1–15. 10.1155/2015/891390 PMC4659957 26640800

[7] Clark RA, Bryant AL, Pua Y, McCrory P, Bennell K, Hunt M. Validity and reliability of the Nintendo Wii Balance Board for assessment of standing balance. Gait Posture. 2010; 31: 307–10. 10.1016/j.gaitpost.2009.11.012 20005112

[8] Glatthorn JF, Gouge S, Nussbaumer S, Stauffacher S, Impellizzeri FM, Maffiuletti NA. Validity and reliability of Optojump photoelectric cells for estimating vertical jump height. J Strength Cond Res. 2011; 25(2): 556–60. 10.1519/JSC.0b013e3181ccb18d 20647944

[9] Clark RA, Mentiplay BF, Pua YA, Bower KJ. Reliability and validity of the Wii Balance Board for assessment of standing balance: A systematic review. Gait Posture. 2018; 61: 40–54. 10.1016/j.gaitpost.2017.12.022 29304510

[10] Palmieri RM, Ingersoll CD, Stone MB, Krause BA. Center-of-pressure parameters used in the assessment of postural control. J Sport Rehabil. 2002; 11: 51–66.

[11] Salavati M, Hadian MR, Mazaheri M, Negahban H, Ebrahimi I, Talebian S., et al Test-retest reliability of center of pressure measures of postural stability during quiet standing in a group with musculoskeletal disorders consisting of low back pain, anterior cruciate ligament injury and functional ankle instability. Gait Posture. 2009; 29(3): 460–4. 10.1016/j.gaitpost.2008.11.016 19167891

[12] Linthorne NP. Analysis of standing vertical jumps using a force platform. American Journal of Physics. 2001; 69: 1198–204.

[13] Markovic G, Dizdar D, Jukic I, Cardinale M. Reliability and factorial validity of squat and countermovement jump tests. J Strength Cond Res. 2004; 18(3): 551–5. 10.1519/1533-4287(2004)18<551:RAFVOS>2.0.CO;2 15320660

[14] Gil-Gomez JA, Llorens R, Alcaniz M, Colomer C. Effectiveness of a Wii balance board-based system (eBaViR) for balance rehabilitation: a pilot randomized clinical trial in patients with acquired brain injury. Journal of NeuroEngineering and Rehabilitation. 2011; 8: 30–8. 10.1186/1743-0003-8-30 21600066 PMC3120756

[15] Young W, Ferguson S, Brault S, Craig C. Assessing and training standing balance in older adults: a novel approach using the ‘Nintendo Wii’ balance board. Gait Posture. 2011; 33: 303–5. 10.1016/j.gaitpost.2010.10.089 21087865

[16] Franco JR, Jacobs K, Inzerillo C, Kluzik J. The effect of the Nintendo Wii Fit and exercise in improving balance and quality of life in community dwelling elders. Technology and Health Care. 2012; 20(2): 95–115. 10.3233/THC-2011-0661 22508022

[17] Nilsagard YE, Forsberg AS, Koch L. Balance exercise for persons with multiple sclerosis using Wii games: a randomised, controlled multi-centre study. Multiple Sclerosis Journal. 2013; 19(2): 209–216. 10.1177/1352458512450088 22674972

[18] Toulette C, Toursel C, Oliver N. Wii Fit training vs. Adapted Physical Activities: which one is the most appropriate to improve the balance of independent senior subjects? A randomized controlled study. Clin Rehabil. 2012; 26(9): 827–35. 10.1177/0269215511434996 22324055

[19] Koslucher F, Wade MG, Nelson B, Lim K, Chen FC, Stoffregen TA. Nintendo Wii Balance Board is sensitive to effects of visual tasks on standing sway in healthy elderly adults. Gait Posture. 2012; 36: 605–8. 10.1016/j.gaitpost.2012.05.027 22748469

[20] Yamamoto K, Matsuzawa M. Validity of a jump training apparatus using Wii Balance Board. Gait Posture. 2013; 38: 132–5. 10.1016/j.gaitpost.2012.11.002 23219781

[21] Jeter PE, Wang J, Gu J, Barry MP, Roach C, Corson M, Yang L, Dagnelie G. Intra-session test-retest reliability of magnitude and structure of center of pressure from the Nintendo Wii Balance Board for a visually impaired and normally sighted population. Gait Posture. 2015; 41(2): 482–7. 10.1016/j.gaitpost.2014.11.012 25555361 PMC4385439

[22] Bartlett HL, Ting LH, Bingham JT. Accuracy of force and center of pressure measure of the Wii Balance Board. Gait Posture. 2014; 39(1): 224–18. 10.1016/j.gaitpost.2013.07.010 23910725 PMC3842432

[23] Donath L, Roth R, Zahner L, Faude O. Testing single and double limb standing balance performance: Comparison of COP path length evaluation between two devices. Gait Posture. 2012; 36(3): 439–43. 10.1016/j.gaitpost.2012.04.001 22565319

[24] Scoppa F, Capra R, Gallamini M, Shiffer R. Clinical stabilometry standardization: basic definitions-acquisition interval-sampling frequency. Gait Posture. 2013; 37: 290–2. 10.1016/j.gaitpost.2012.07.009 22889928

[25] Bland JM, Altman DG. Statistical methods for assessing agreement between two methods of clinical measurement. Lancet. 1986; 1(8476): 307–10. 2868172

[26] Larsen LR, Jörgensen MG, Junge T, Juul-Kristensen B, Wedderkopp N. Field assessment of balance in 10 to 14 year old children, reproducibility and validity of the Nintendo Wii board. BMC Pediatr. 2014; 14(1): 144. 24913461 10.1186/1471-2431-14-144 PMC4057805

[27] Bonnechère B, Jansen B, Omelina L, Van Sint Jan S. The use of commercial video games in rehabilitation: a systematic review. Int J Rehabil Res. 2016; 39(4): 277–90. 10.1097/MRR.0000000000000190 27508968

[28] Bower KJ, McGinley JI, Miller KJ, Clark RA. Instrumented static and dynamic balance assessment after stroke using Wii Balance Boards: reliability and association with clinical test. PLoS One. 2014; 9(12):e115282 10.1371/journal.pone.0115282 25541939 PMC4277284

[29] Llorens R, Latorre J, Noé E, Keshner EA. Posturography using the Wii Balance Board. A feasibility study with healthy adults and adults post-stroke. Gait Posture. 2016; 43: 228–32. 10.1016/j.gaitpost.2015.10.002 26584877

[30] Holmes JD, Jenkins ME, Johnson AM, Hunt MA, Clark RA. Validity of the Nintendo Wii balance board for the assessment of standing balance in parkinson’s disease. Clin Rehabil. 2013; 27(4): 361–6. 10.1177/0269215512458684 22960241

[31] Doyle TL, Newton RIJ, Burnett AF. Reliability of traditional and fractal dimension measures of quiet stance center of pressure in young, healthy people. Arch Phys Med Rehabil. 2005; 86(10): 2034–40. 10.1016/j.apmr.2005.05.014 16213250

[32] Castelli I, Stocchi L, Patrignani M, Sellitto G, Giuliani M, Prosperini L. We-Measure: Toward a low-cost portable posturography for patients with multiple sclerosis using the commercial Wii Balance Board. J Neurol Sci. 2015; 359(1–2): 440–4. 10.1016/j.jns.2015.10.016 26490321

[33] Hubbard B, Pothier D, Hughes C, Rutka J. A portable, low-cost system for posturography: a platform for longitudinal balance telemetry. J Otolaryngol Head Neck Surg. 2012; 41: S31–5. 22569047

[34] Park DS, Lee G. Validity and reliability of balance assessment software using the Nintendo Wii Balance Board: usability and validation. J Neuroeng Rehabil. 2014; 11: 99 10.1186/1743-0003-11-99 24912769 PMC4074461

[35] Dickinson JI, Shroyer JL, Elias JW. The influence of commerical-grade carpet on postural sway and balance strategy among older adults. Gerontologist. 2002; 42(4): 552–9. 10.1093/geront/42.4.552 12145383

[36] Owen NJ, Watkins J, Kilduff LP, Bevan HR, Bennett MA. Development of a criterion method to determine peak mechanical power output in a countermovement jump. J Strength Cond Res. 2014; 28: 1552–8. 10.1519/JSC.0000000000000311 24276298

[37] Huurnink A, Fransz DP, Kingma I, van Dieën JH. Comparison of a laboratory grade fore platform with Nintendo Wii Balance Board on measurement of postural control in single-leg stance balance tasks. J Biomech. 2013; 46(7): 1392–5. 10.1016/j.jbiomech.2013.02.018 23528845

[38] Pagnacco G, Oggero E, Wright CH. Biomedical instruments versus toys: a preliminary comparison of force platforms and the nintendo wii balance board. Biomed Sci Instrum. 2011; 47: 12–7. 21525589

